# Delayed periprosthetic seroma in a male Poland syndrome patient: A case report

**DOI:** 10.1097/MD.0000000000024974

**Published:** 2021-03-12

**Authors:** Jangyoun Choi, Jung Hyeou Kim, Yeoun Eun Sung, Deuk Young Oh

**Affiliations:** a Department of Plastic and Reconstructive Surgery; b Department of Hospital Pathology, Seoul St. Mary's Hospital, The Catholic University of Korea, College of Medicine, Seoul, Korea.

**Keywords:** Breast implant associated – anaplastic large cell lymphoma, chest wall deformity, customized implant, delayed seroma, Poland syndrome

## Abstract

**Rationale:**

Custom-made implant is an accepted treatment option for treatment of chest deformity in Poland syndrome. Unlike the raised concerns and awareness for the long-term consequences of breast implants, the long-term complications of customized implants for special purposes like Poland syndrome has not been reported in the literature.

**Patient Concerns:**

A 44-year-old male with Poland syndrome presented to our institution complaining of a large bulge and fluctuation on the right chest wall. This occurred after 14 years from the initial implant surgery for correction of chest wall deformity. Upon failure of resolution by multiple aspirations, workup was carried out under suspicion of implant associated malignancy.

**Intervention:**

Total Capsulectomy and implant removal was done.

**Outcomes:**

Histology revealed chronic inflammation with fibrosis. Implant-associated malignancy was not found. He is being followed up with no signs of recurrence.

**Lessons:**

For rare cases of implant insertion such as Poland syndrome, awareness of delayed complications and workups based on suspicion of implant-associated malignancy is needed. Surgeon awareness and patient education is required.

## 1 Introduction

Chest reshaping in male Poland syndrome patient is an infrequent clinical experience.^[1]^ Due to its rarity, its long-term clinical consequence has almost never been reported.^[2]^ Despite the rising concerns of implant-associated complication and potential malignancy, accumulation of clinical experience in this small patient group is an unmet need.

Compared to autologous reconstruction, deformity correction with customized implant is a feasible option especially for male patients when they require preservation of muscle function, particularly the latissimus dorsi muscle. Although implant-related complications do not show gender predilection, late periprosthetic fluid collection and capsule formation in a male patient has seldom been reported in the literature. Here we report a male Poland syndrome patient with delayed periprosthetic fluid collection which showed multiple relapses despite clinic aspirations. *En-bloc* surgical removal of the capsule and implant was undertaken. Cytology and biopsy showed negative results for ALCL-related markers. Tips and pearls for office-based management and surgery, with literature review is presented.

## 2 Case presentation

A 44-year-old man was referred to our institution for evaluation of bulging on his right chest wall. Bulging had started spontaneously without any event of trauma to the chest wall. On history, he had received silicone prosthesis insertion 14 years ago for correction of Poland syndrome at a different institution. Except for the chest wall, he did not show additional deformities related to Poland syndrome, such as hand or upper arm hypoplasia. Otherwise his medical history was unremarkable.

**Figure 1:**
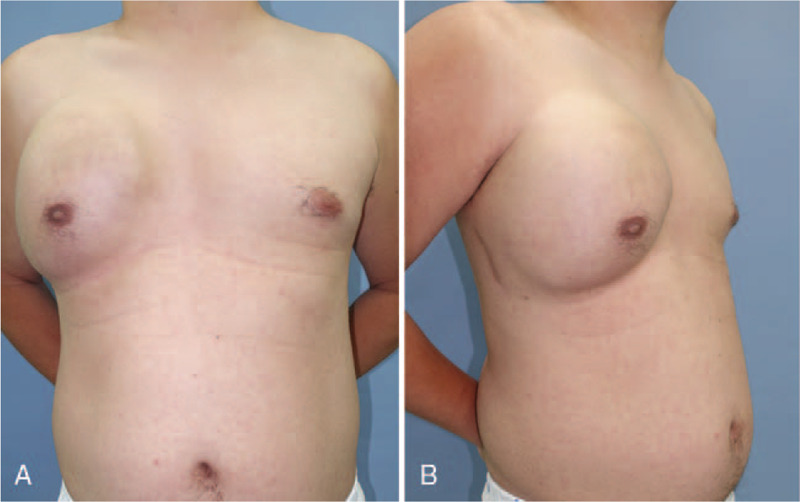
Clinical photo of the patient at initial presentation. Unilateral bulging of the right pectoral area is noted. Left, frontal view. Right, Oblique view.

**Figure 2:**
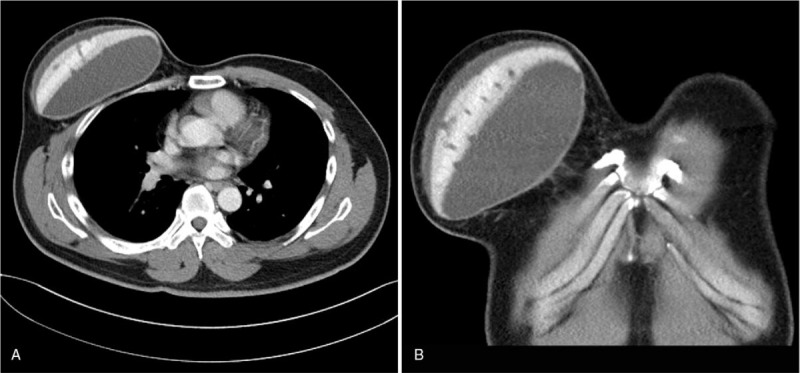
Radiologic finding of the patient. Left, Pectoral muscle is not found on the right side. About 4 mm-thickness rim enhancement under the subcutaneous tissue is noted, suggesting prominent capsule formation. Internal medium signal intensity suggests a large amount of fluid collection. Inside the capsule, the custom-made implant is noted. Right, Coronal view of the CT, showing the largest diameter of the implant measureing over 16 cm. Evenly spaced punched-out lesions are found, which was presumed to be the result of customization.

On physical exam, fluctuation and slight erythema was persistent over the whole right chest wall.(Fig. 1) Radiologic exam was performed for baseline evaluation.(Fig. 2) CT revealed absence of right pectoralis muscle, and a subcutaneous dome-shaped attenuation measuring 16.2 cm in its base diameter, highly suggesting periprosthetic fluid collection. The fluid was seen on both the superficial and deep plane of the prosthesis. Inside the prosthesis, numerous full-thickness perforations of regular diameter were found, which explained the massive fluid collection on both planes around the prosthesis. Under the impression of late periprosthetic seroma formation, syringe aspiration was done. About 500CC of serous fluid was evacuated and sent out for cytology. Cytology showed mixed inflammatory cells which was consistent with chronic active inflammation. Immunohistochemistry for CD30 showed negativity. However, fluid collection relapsed and required additional aspirations. After 2 rounds of office-based aspiration, surgical management with implant removal and complete capsulectomy was planned.

**Figure 3:**
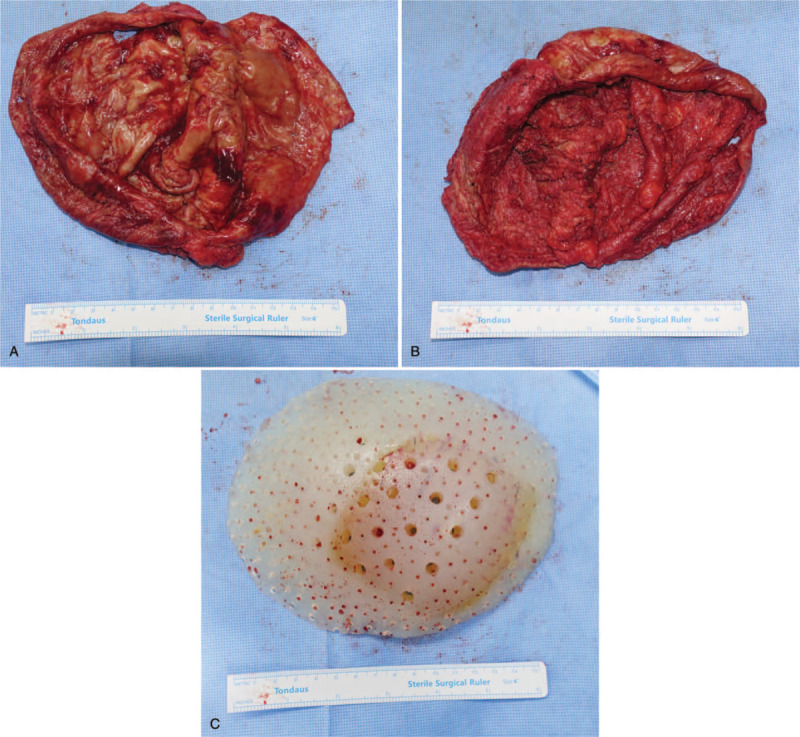
Gross specimen of the explant and the surrounding capsule. (Left) Outer aspect. (Center) Inner aspect. (Right) Explanted prosthesis.

Under general anesthesia, a 6 centimeter-sized oblique incision was designed slightly above the right costal margin. Upon incision and subcutaneous dissection, serous fluid was drained. After evacuation of remnant fluid, capsule was revealed. The capsule and implant was removed *en-bloc* with minimal injury. (Fig. 3) Gross examination showed a very thick capsule, measuring over 4 millimeters in thickness. Multiple, atypical nodular growth over the whole implant capsule was found. The capsule was sent out for histology. The pocket was thoroughly irrigated and closed with negative suction drains. Postoperative course was uneventful.

**Figure 4:**
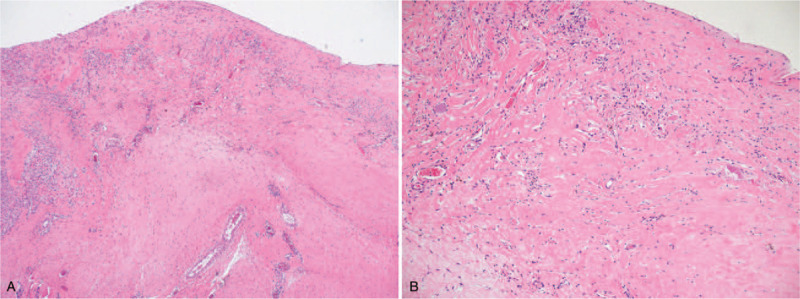
Histologic analysis of the capsule. (Left) The nodular growth which was an atypical gross feature of the capsule was found to be benign. (Right) Overall, benign-looking accumulation of inflammatory cells without atypia, and collagen deposition is found. (H&E stain, ×40 and ×100 magnification.).

**Figure 5:**
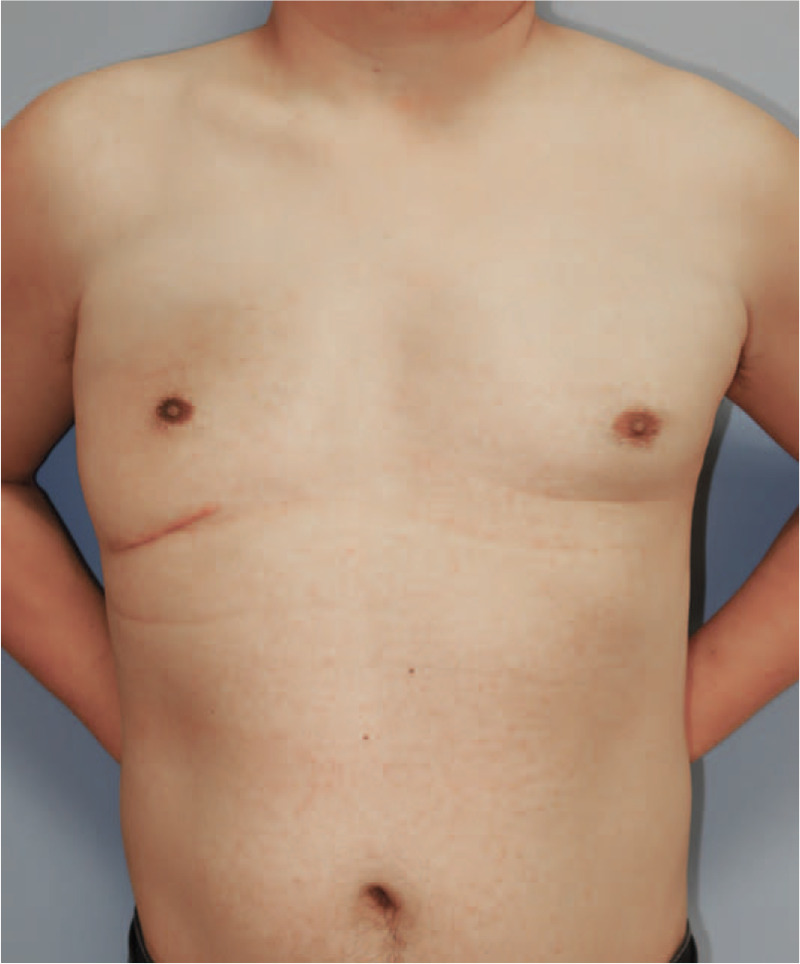
Follow-up photo after 1 year from surgery.

Histology revealed chronic inflammation with fibrosis. Fortunately, no evidence of implant-associated malignancy was found. (Fig. 4) Up to 1 year, He is being followed up with no signs of recurrence. (Fig. 5) Informed written consent was obtained from the patient for publication of the images.

## 3 Discussion

Poland syndrome is a group of congenital anomalies in chest wall and upper limb that shows wide clinical variability.^[3]^ Its clinical spectrum spans from mild asymmetry of chest wall to severe upper limb deformity, or even absence of visceral organs such as the kidney. Its cause is unclear, but circulatory underdevelopment to the chest and upper limb is accepted as the most plausible pathophysiology.^[1]^ Among the clinical features, deficient pectoral muscles and resultant asymmetry is the hallmark finding.

Unlike aesthetic or reconstructive breast surgery where premanufactured silicone gel implants are the norm, custom-made implants are used in custom-made implants are implemented for treatment of chest asymmetry in Poland syndrome. This is especially feasible in physically active male patients when autologous reconstruction is unfavored due to the desire for preserving donor muscle function.^[4]^

Although the reason is not clear, previous literature state low long-term complication rates. The most recent and large-volume research is by Chavoin in 2018, who reviewed their patients treated for Poland syndrome with customized implants. They reported no occurrence of long-term capsular contractures in custom made chest implants.^[5]^ Another report by Pereira et al also reported absence of capsular contracture in prosthetic correction of male Poland syndrome patients for 4 years of follow up.^[4]^ However, our case suggests the need for a longer and closer follow up for development of delayed complications in customized implant patients, just as the conventional silicone breast implants used for female breasts.

Chronic inflammation plays a crucial role in developing capsular contracture and also implant-related malignancy, namely Breast implant associated – anaplastic large cell lymphoma. Therefore, we think cytological analysis was a crucial step in workup of our case, even though the results were benign. Current guidelines suggest aspiration of at least over 50cc and sent for cytology to assess cell morphology and CD30 immunihistochemistry.^[6]^

Gross finding of the capsule is a noteworthy feature of this case. A much thicker capsule was found compared with usual breast implant capsules, which was measured over 4 millimeters. Moreover, multiple, solid, and mass-like growth was found over the whole capsule, which is a very distinct feature compared with the conventional breast implant capsules. It is not clear whether this was just related to the customized property of the implant, or a feature indicating progressive change towards malignancy. In either situation, this case illustrates the need for the surgeon to take a thorough gross inspection of the capsule after capsulectomy. After inspection, a proactive inform to the pathologist for raised suspicion would be highly desirable.

When performing capsulectomy, we strongly recommend attempting total capsulectomy at all times. Since the malignancy develops in a localized area in the capsule, partial capsulectomy has the risk of leaving behind the portion at risk of malignancy. Therefore, we recommend using the IMF incision to achieve complete visualization of the implant pocket and perform total capsulectomy. In our case, the patient had received his initial implant insertion through a lateral thoracic incision. However, we did not hesitate to approach through a new inframammary incision to clearly visualize the capsule and evacuate it. Furthermore, the use of ultrasound-generated harmonic scalpel is a useful adjunct to clearly delineate the capsule-tissue interface without bleeding.^[7]^ It is our routine practice to attempt total capsulectomy at all times, and this is most always possible with the aid of the harmonic scalpel.

Delayed, implant-related complications can arise in any patient group who received implant insertion, regardless of gender, or the underlying cause for augmentation. Although customized implants are used infrequently, they need the same level of attention as the conventional silicone breast implants. Male Poland syndrome patients who received implant insertion are usually incognizant of the risk they might possess. It is advisable to educate patients for long-term complications of implants. Cytology analysis of the fluid collection should be undertaken at all times, including immunohistochemistry for ALCL-related markers. A total capsulectomy and implant removal is indicated if the presenting symptom such as fluid collection does not resolve despite aspiration.

## Author contributions

**Conceptualization:** Deuk Young Oh.

**Data curation:** Jangyoun Choi, Jung Hyeou Kim, Yeoun Eun Sung, Deuk Young Oh.

**Visualization:** Jangyoun Choi, Jung Hyeou Kim, Yeoun Eun Sung.

**Writing – original draft:** Jangyoun Choi.

**Writing – review & editing:** Deuk Young Oh.
